# Implementation of an early attention strategy to reduce emergency room overcrowding in an academic institution in Colombia, a pilot study

**DOI:** 10.1186/s12245-024-00700-7

**Published:** 2024-10-10

**Authors:** German Devia Jaramillo, Nathalia Esmeral Zuluaga, Viviana Andrea Velandia Avellaneda, Salvador Menendez Ramirez, Fernando Jose Pimienta Neira, Angie Paola Lopez Contador, Juan Pablo Vargas Gallo

**Affiliations:** 1 Fundación Santafé de Bogotá. Grupo de Investigación Clínica, Escuela de Medicina y Ciencias de La Salud, Universidad del Rosario, Bogotá, Colombia; 2 School of Medicine and Health Sciences, Universidad del Rosario, Bogotá, Colombia; 3 Fundación Santafé de Bogotá, Universidad de los Andes, Bogotá, Colombia; 4 Fundación Santafé de Bogotá, Universidad del Rosario, Bogotá, Colombia; 5 Fundación Santafé de Bogotá, Bogota, Colombia

**Keywords:** Patient satisfaction, Emergency department, Overcrowding, Crowding interventions, Crowding solutions

## Abstract

Overcrowding is a worldwide problem, and long waiting times are associated with increased morbidity and even mortality of patients regardless of triage classification. Although there are many tools published in the literature that contribute to the reduction of overcrowding, for the Colombian population there are not many tools evaluated to reduce the length of stay of patients in the emergency department. This is a retrospective analytical study that compared whether there was a difference in patient definition time and ED length of stay between a group attended under an early care protocol (PAT) versus the usual protocol. Of the total of 969 patients included it was found that the group attended under the PAT protocol had a shorter definition time than the usual protocol, also the Emergency department length of stay (EDLOS) was significantly lower in the PAT group compared to the usual protocol. The implementation of the PAT protocol performed by emergency physicians allows a faster contact with the patient by the physician, and leads to a significant reduction of EDLOS, contributing to the reduction of overcrowding in the emergency department.

## Introduction

Waiting time for ED patients is a problem for all healthcare systems worldwide [1–4]. In addition, prolonged length of stay in the emergency department, described in the literature as Emergency department length of stay (EDLOS), is associated with increased morbidity and mortality [5, 6]. This problem, being so complex, requires multiple strategies for its resolution, so that several alternatives can be found in the scientific literature [7–10]. Nevertheless, there are not many tools evaluated to reduce EDLOS and emergency room overcrowding for the Colombian population.

This is why this study aims to determine how the flow of patients attending the hospital classified as triage 3 emergencies will behave with the implementation of an early care protocol (PAT), compared to the usual care protocol.

## Methods

### Study design

This is a retrospective analytic observational study.

### Population

The study used information from the emergency department databases of the Hospital Universitario Fundación Santafé. This highly complex university institution attends approximately 60,000 adult emergencies of which approximately 40% are medium complexity emergencies classified as triage 3.

### Eligibility criteria

Adult patients attending the Hospital Universitario Fundación Santafé for emergency medical care classified as triage 3 emergencies, who were evaluated under the usual care protocol and PAT, were included in the study, which was carried out between August and October 2023. Pregnant patients, population under 18 years of age, orthopedic emergency patients and incomplete data in the medical records were excluded.

### Methodology

Patients seen in October were the intervention group while patients seen in the month of August were considered the comparison group. The hypothesis of this work was that with the proposed intervention in triage, it could contribute to the reduction of overcrowding of the emergency department, by reducing the length of stay in the emergency room, definition times and total time of attention. Initially the PAT (Physician at Triage), protocol was implemented in October between 10 am to 7 pm from Monday to Friday for patients with triage 3 classification, for this reason the patients who were classified in this same way during the previously mentioned hours in August were selected to make a proper comparison.

### PAT Protocol

Patients who come for emergency medical attention to the emergency department of the institution are classified in triage by a group of trained nurses, patients classified as triage 3 were admitted to the PAT room consisting of two cubicles with a chair for the patient in which a trained emergency physician did a short interview, In addition, the emergency physician could refer the patient to the resuscitation room in the event of finding a patient with signs of a serious illness.

Each assessment by the emergency physician in the PAT cubicle lasted no more than 10 min. Once the patient was assessed by this specialist, orders for the pertinent studies and interventions were given and carried out by two nurses assigned for this purpose.

Subsequently, the patient was called to the general consultation room, the “Box” room, by another emergency physician, where the complete medical consultation was performed and the studies and interventions carried out in the PAT room could be interpreted. From the Box room, patients could be discharged or left in hospitalization, some few were taken for further studies before the emergency physician defined the course of action.

### Time definition

The following time definitions were used for the development of this work:

Time to Triage: is the time in minutes elapsed between the patient's request for care through an electronic shift and the initial assessment performed in the triage room of the emergency department by the nurse in charge of initial patient triage.

Time Triage to PAT Doctor: is the time in minutes elapsed after the initial patient triage by the triage nurse and the first contact with the emergency physician; this time for the intervention group was considered as the time for the initial contact with the physician of the PAT protocol.

Time Triage to Consultation: is the time measured from the triage nurse's assessment to the patient's first contact with the emergency physician; for the comparison group the time to Doctor and the time to consultation was the same, while for the intervention group, the time to consultation is the time elapsed between the initial assessment by the triage nurse and the formal consultation by the emergency physician in the consultation room or Box room. Time to definition: is the time elapsed from the initial assessment by the emergency physician to the definition of disposition in the emergency department, this disposition could be discharge or hospitalization. In the PAT group is taken from the beginning of the consultation in the BOX area, not from the first contact with the doctor, since this is not considered a consultation. In short this is the time to the decision to discharge or refer for admission. Boarding Time: is the time elapsed from the hospital admission order made by the physician and the physical transfer of the patient to the hospitalization floors of the institution.

EDLOS: is the total length of stay of patients in the emergency department, from the start of triage by the nurse to the definition of definitive behavior by the emergency physician.

### Sample size

All patients seen with emergencies classified as triage 3 during the months who met the inclusion criteria were included. Since this was a pilot observational study, no sample size calculation was performed.

### Data analysis plan

Differences in age, number of patients, gender, admission diagnosis were evaluated to establish comparability between groups. For quantitative variables, differences were established by calculating Student's t-test (if the variable had normal distribution), or Wilcoxon (if it did not have normal distribution). The chi2 test was used to determine differences in categorical variables.

A descriptive analysis of the variables under study was performed, with frequency distribution for categorical variables and central tendency and dispersion measurements for continuous variables according to the type of distribution (mean and standard deviation or median and interquartile ranges (IQR) for normal or non-normal distribution, respectively), for which the normality test (Kolmogorov–Smirnov) was applied.

Subsequently, statistical significance was established for the mean differences in the values of waiting time to triage, time to contact with the physician, observation time, definition time, boarding time, and total ED length of stay (EDLOS) using Student's t test for normally distributed variables or Wilcoxon for non-normally distributed variables. In the case of comparing categorical variables, these were evaluated using the chi2 test. The statistical program R Version 1.4.1106 © 2009–2021 RStudio, PBC was used for the analyses.

## Results

**Fig. 1:**
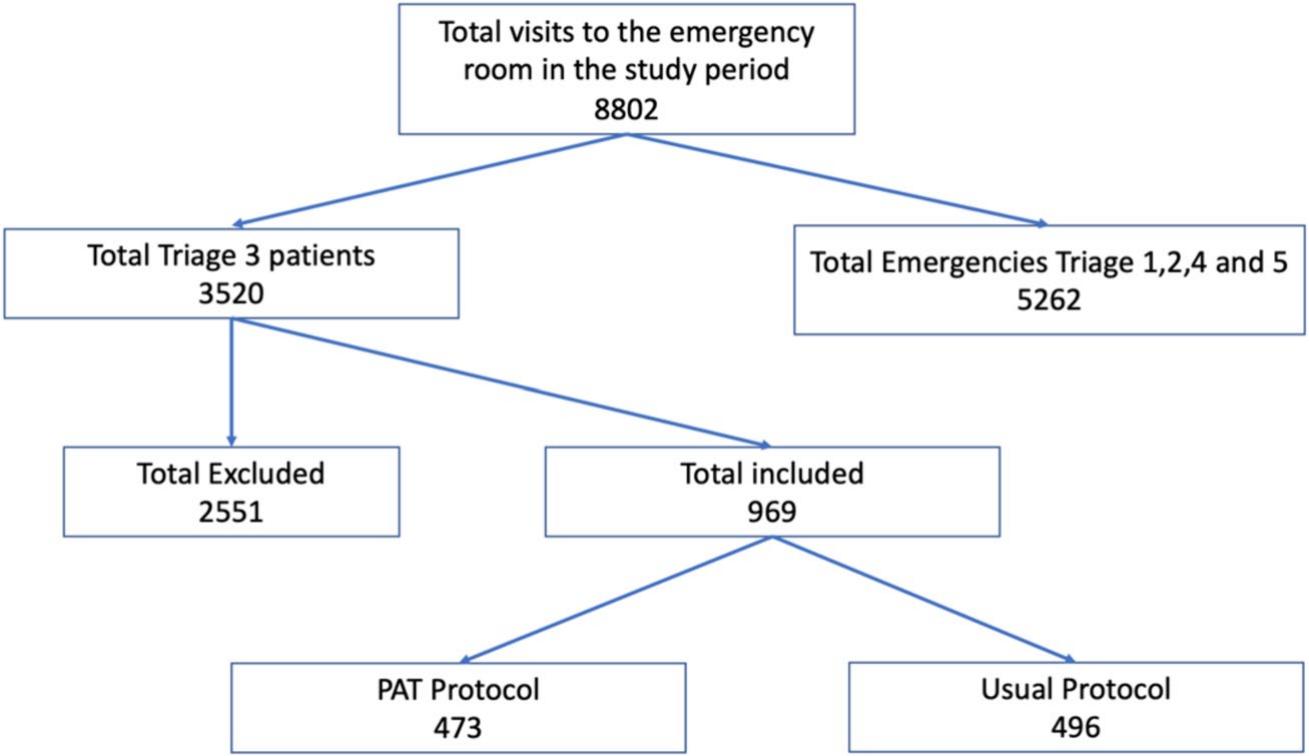
Patient selection algorithm

A total of 969 patients were collected during the study period, of which 473 entered the PAT protocol. See Fig. 1.

The mean age of the patients was 57 years (IQR 18–101), 40.7% were men and the most frequent diagnosis was infectious pathology (28.6%).

It was also documented that there was no significant difference in the time to triage, i.e. the time since the patient requested care and the time to the first assessment by the triage nurse Table 1.

**Table 1:** Patient characteristics

	PAT Protocol	Usual Protocol	*P* value
n	473	496	
Age	54(36–73)	59(41–74)	0.017
Female sex (%)	268(65.7)	307(61.9)	0.111
Diagnosis group (%)			0.283
Infectious	124(26.2)	153(30.9)	
Abdominal	98(20.7)	96(19.4)	
Cardiovascular	51(10.8)	65(13.1)	
Hematoncological	37(7.8)	23(4.6)	
Neurological	37(7.8)	36(7.3)	
Time to triage Min Median(IQR) Median(IQR)	32(18–45)	30(17–43)	0.262

Regarding the main outcomes, it was documented that the PAT group had a slight decrease in the time for the physician's initial contact with the patient with respect to the usual protocol, although this difference was not statistically significant. Likewise, it was found to be statistically significant that the PAT group had a longer waiting time to be evaluated in the formal consultation of the emergency physician with respect to the group attended by the usual protocol, despite this, the group attended by the PAT protocol was defined more quickly than the patients in the usual group; this definition was reflected in a significant reduction in the total length of stay in the emergency department of the patients attended by the PAT protocol with respect to the patients attended by the usual protocol.

Finally, no differences were found in Boarding time between the two groups. Table 2.

**Table 2:** Comparison of times between the two groups

	**Protocolo PAT**	**Protocolo Usual**	*P* value
Time to Doctor Min (median IQR)	70(42–109)	69.7(42.3–140.2)	0.149
Time to consultation Min (median IQR)	242(170.5–312)	69.72(42.3–140.2)	< 0.001
Time to definition Min (median IQR)	38(1–252)	208(1–416)	< 0.001
Boarding time(median IQR)	0(0–157.8)	0(0–298.9)	0.089
EDLOS Min (median IQR)	440(318.3–840.9)	523.5(318–1379)	0.037

## Discussion

The objective of this study was to evaluate the usefulness in reducing overcrowding of an early care strategy for patients consulting the emergency department classified as triage 3 in a level 3 academic institution in Colombia. This study that recruited 969 patients to analyze. This pilot study demonstrated that with the use of a physician performing advanced triage, the length of stay of patients in the emergency department was reduced, as well as the definition time. This could be attributed to the fact that the doctors, when consulting with the patients, already had sufficient diagnostic tools to make the decision on the definitive conduct with the patients. It was considered that the groups were comparable despite the difference in age, since no differences were demonstrated between groups in the time to initial triage assessment, in the sex of the patients, the diagnoses between groups and a similar number of subjects representing each group was obtained.

There are strategies in the literature for early care of patients who come to the emergency department, such as advanced triage performed by nurses trained in special situations such as minor trauma [11] which showed a decrease in care time from 55 min to 12.8 min, initiation of pain management [12] in which the time for the administration of analgesia was reduced to 25 min and 61.3% of patients received analgesia. 3% of patients received analgesia in less than 30 min after assessment, minor fractures [13] where no difference in diagnosis and management was documented between trained nurses compared to senior house officer doctors, uncomplicated urinary tract infection [14] where no difference in antibiotic prescription was documented between trained nurses and physicians, non-traumatic chest pain [15] where no difference was found between length of stay (LOS) and waiting times between patients assessed by trained nurses and the standard model. However, there are not many publications on the model proposed in this study, in which it is not an advanced triage performed by a nurse, but a “preconsultation” performed by the emergency physician after conventional triage.

One of the objectives of this protocol was to achieve earlier contact between the physician and the patient; however, although the study showed a tendency to reduce the time for this contact, this time was not statistically significant when compared with the usual management. This could be explained by the amount of demand for consultation and the care staff established during the hours in which the work was performed. Likewise, the present study showed that even the time to consultation by the emergency physician in the intervention group was statistically longer compared to the usual protocol, it is possible that this delay was due to fact that the emergency physicians in the box room were awaiting results before seeing the patients. Although there was already a previous physician–patient contact, and this time was similar in the two groups.

The delay in the documented care is compensated by the time of patient definition, in the intervention group this time was statistically lower compared to the comparison group, this time is reduced since the emergency physician in the Box room already had reports of diagnostic studies, additionally he could evaluate the evolution of some minimally invasive interventions performed in the PAT room, in this way the decision of discharge or admission of the patients was quicker compared to the usual group.

This early care strategy significantly demonstrated a reduction in EDLOS, there are few studies that have achieved this reduction with interventions close to triage, the work of Tucker [16], showed a decrease of 18 min in LOS when a trained nurse evaluated patients with minor emergencies from triage, similar to the work of Garder et al. [17] where this type of care to patients with minor emergencies decreased LOS by 34 min, the study of Colligan et al. [18] in patients with minor trauma decreased LOS by 40 min. These studies, as well as the one reported in this document, show that it is possible to reduce the length of stay of patients in the emergency room, thus contributing to the reduction of overcrowding.

This work did not show a difference in boarding time, which is expected given that this time does not depend only on the consultation and the flow of the emergency department, it also depends on the hospital flow per se, which largely explains the complexity of finding significant solutions to overcrowding.

With the results of this pilot study, it was possible to demonstrate that the implementation of an early care strategy close to triage by emergency physicians contributes in some way to the solution of overcrowding in the emergency department; however, a more robust study is needed to determine the applicability of this process in other emergency departments.

## Limitation

This study has numerous limitations, the first being that it is a retrospective study, except that the times and other variables evaluated were obtained from the institution's information system, which makes this information more reliable.

This is a pilot study without a sample size calculation; however, the results of this work allow us to continue with the implementation of the early care protocol in a larger population.

The PAT was implemented during specific hours of the day, which could have some bias since at night or on weekends the ED congestion could change, but likewise the comparison group was also obtained during the same hours to have similar conditions of care.

The intervention group was younger, it is possible that older patients may require more studies and additionally have a greater probability of hospital admission, this could have influenced the results of the study. However, we do not consider that the age difference was so high as not to validate the importance of the results obtained. The diagnoses were also evaluated which were similar between groups and the initial time to triage assessment was not different between the compared groups.

## Conclusion

The implementation of the PAT protocol performed by emergency physicians allows a faster contact with the patient by the physician, and significantly allows a faster patient definition than the usual protocol of care and leads to a significant reduction of EDLOS, contributing to the reduction of overcrowding in the emergency department.

## Data Availability

No datasets were generated or analysed during the current study.
